# An Environment-Sensitive Synthetic Microbial Ecosystem

**DOI:** 10.1371/journal.pone.0010619

**Published:** 2010-05-12

**Authors:** Bo Hu, Jin Du, Rui-yang Zou, Ying-jin Yuan

**Affiliations:** Key Laboratory of Systems Bioengineering, Ministry of Education and Department of Pharmaceutical Engineering, School of Chemical Engineering and Technology, Tianjin University, Tianjin, People's Republic of China; Center for Genomic Regulation, Spain

## Abstract

Microbial ecosystems have been widely used in industrial production, but the inter-relationships of organisms within them haven't been completely clarified due to complex composition and structure of natural microbial ecosystems. So it is challenging for ecologists to get deep insights on how ecosystems function and interplay with surrounding environments. But the recent progresses in synthetic biology show that construction of artificial ecosystems where relationships of species are comparatively clear could help us further uncover the meadow of those tiny societies. By using two quorum-sensing signal transduction circuits, this research designed, simulated and constructed a synthetic ecosystem where various population dynamics formed by changing environmental factors. Coherent experimental data and mathematical simulation in our study show that different antibiotics levels and initial cell densities can result in correlated population dynamics such as extinction, obligatory mutualism, facultative mutualism and commensalism. This synthetic ecosystem provides valuable information for addressing questions in ecology and may act as a chassis for construction of more complex microbial ecosystems.

## Introduction

Microbial ecosystem has increasingly draw our attention for its pivotal roles in the maintenance of Earth's biosphere and sustaining life [1], [2]. It also has great application potential in the production of new-generation bioenergy [3], [4], sewage treatment (Rittman, 2006) and serving as medical targets for intestinal diseases [5]. Numerous researches have shown that interactions between environment and microbial ecosystem largely define structure and scale of ecosystems, but due to complex composition and stochastic population dynamics of natural biological systems, mechanism models uncovering what and how environmental factors influent microbial ecosystem are still waiting to be developed. Although large-scale environmental sequencing has provided information on their structure and functional genes responding to environmental stimulus [6], [7], [8], challenges in coupling system function with ecosystem structure and environmental parameters still exist due to difficulties in quantitative measurement of population dynamics [9]. Thus simplified model system with clear genetic background is necessary for better understanding how microbial ecosystem evolved in different environments.

With the desire to extend natural function and create novel phenotypes by engineering genetic circuits [10]–[17], researchers in the field of synthetic biology had achieved great success in construction of single-cell genetic circuit [18]–[23] and multicellular systems [24], [25], which provides an efficient way to solve above issues by constructing simplified artificial ecosystem with well-defined genetic background and identifiable cellular interactions, such as cooperative yeast community [26], microbial biofilm consortium [27], interspecies symbiosis by air-borne communication [28], *E.coli* prey-predator ecosystem [29] and producer-nonproducer microbial system [30]. Although pioneer advances of synthetic biology proved ecological dynamics could be duplicated on synthetic systems which have many similarities with their natural counterpart, poor understandings of environmental influence extremely constrained its further application. And this issue is what was stressed in the second wave of development of synthetic biology [31].

To better understand how environment and ecosystem interplay with each other, this study designed, simulated and constructed a synthetic symbiosis microbial ecosystem where two *E.coli* populations mutually benefited from each other with the aid of LuxI/R and RhlI/R quorum sensing (QS) signals [32], [33]. Interactions between this synthetic ecosystem and antibiotics, which were normally taken as environmental factors [34], [35], were further investigated.

## Results and Discussion

### The Property of Single Population

**Figure 1:**
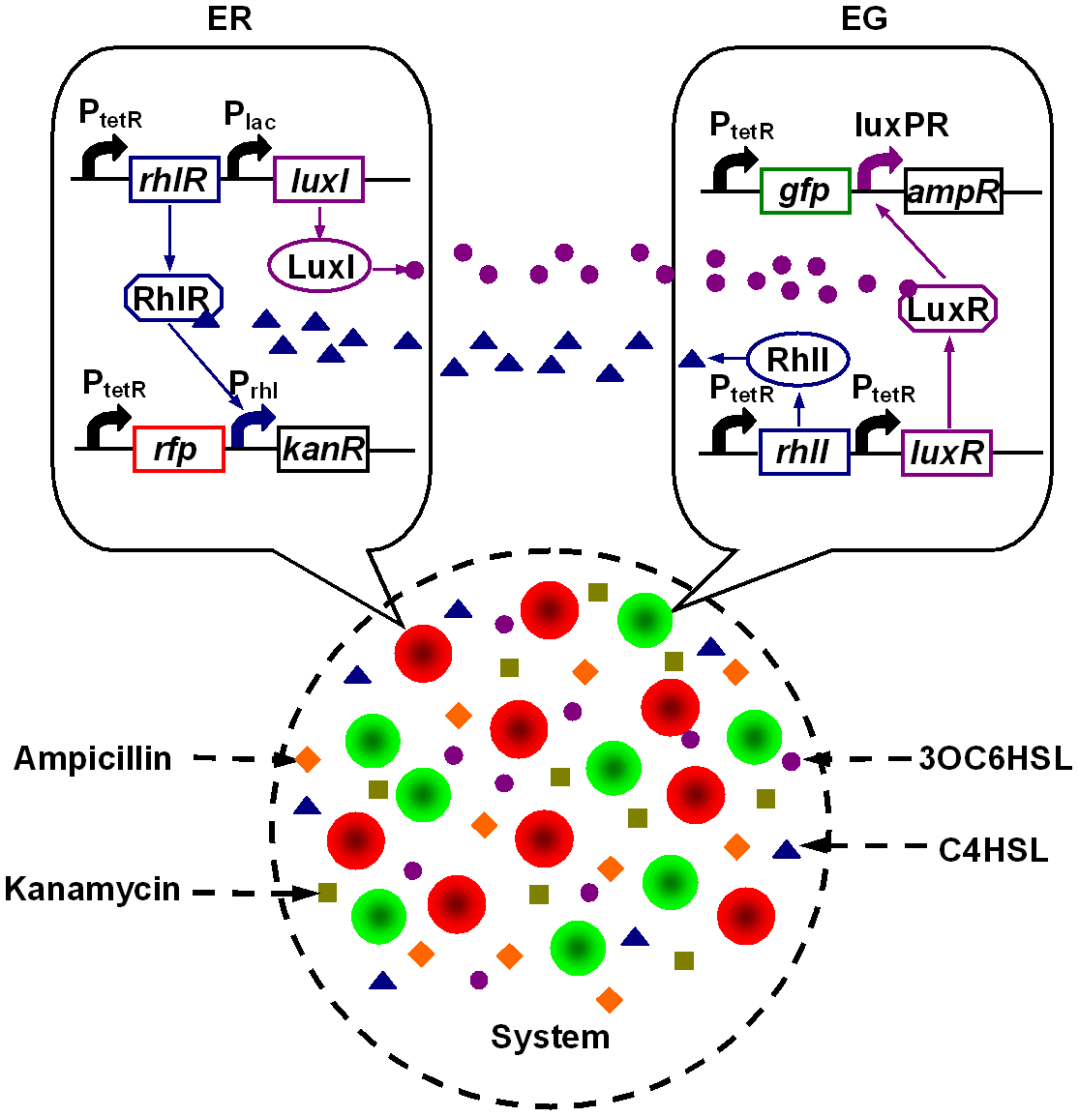
Genetic background of the synthetic microbial ecosystem in this study. Two engineered *E.coli* populations are co-cultured to benefit from each other via two different QS signals. *PtetR* was used as constitutive promoter in our experiments. RFP and GFP under the regulation of *PtetR* were used to distinguish ER and EG by using fluorescence microscope. EG and ER show background resistance to ampicillin and kanamycin respectively by introduction of plasmids PSB1A2 and PSB2K3 where different genetic circuits were organized. RhlR signal receptor was synthesized within ER and could bind with C4HSL signal produced by r*hll* gene in EG cells to activate *Prhl* promoter if the concentration of C4HSL reaches to threshold, which enables ER resistant to kanamycin. Similarly, 3OC6HSL can be synthesized by *luxI* gene in ER cells (controlled by *Plac* promoter, IPTG can be added to increase 3OC6HSL concentration, but even leaky expression can produce enough 3OC6HSL signal), and bind to LuxR signal receptor in EG to initiate *LuxPR* promoter, which makes EG tolerate to ampicillin. Thus individual EG and ER show tolerance to ampicillin and kanamycin respectively due to background vector resistance, while both have double resistance if two cells are co-cultured. RFP and GFP under the regulation of *PtetR* were used to distinguish ER and EG by using fluorescence microscope.

**Figure 2:**
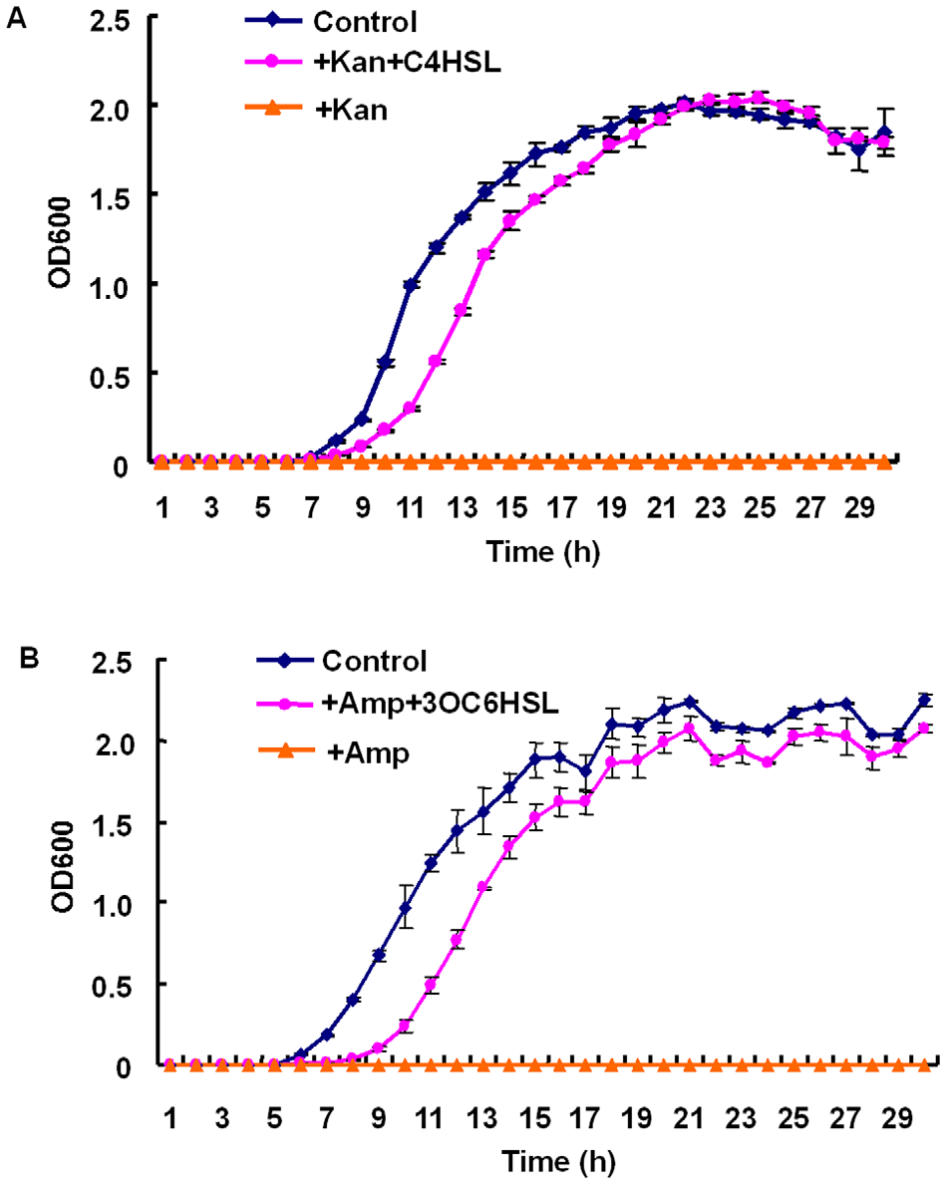
Individual cell function verification. (A) Growth curve (29 hrs) of ER in the medium without kanamycin (solid curve), with kanamycin (1 mg/ml, overlap with x-axis) and with both kanamycin (1 mg/ml) and C4HSL signal (1 µg/ml, dash line). (B) Growth curve (29 hrs) of EG in the medium without ampicillin (solid curve), with ampicillin (5 mg/ml, overlap with x-axis) and with both ampicillin (5 mg/ml) and 3OC6HSL signal (1.1 µg/ml, dash curve). Data are means of triplicate experiments.

Two *E.coli* populations were genetically engineered as shown in Figure 1, which was illustrated in detail in ‘Materials and Methods’ part. And properties of single population were firstly tested. Compared with low kanamycin level (50 µg/ml), high kanamycin concentration (1 mg/ml) significantly inhibited ER's growth (Figure 2). However, ER cells restrained by kanamycin could be rescued by addition of C4HSL (1 µg/ml) which bound to RhlR protein to activate the expression of kanamycin resistance gene in ER. Rescued ER (with kanamycin and C4HSL) grew slightly slower than those in the normal LB medium, possibly due to metabolic burden brought by overexpression of circuit components or the extended lag phase required to degrade kanamycin, or both. Similarly, EG failed to grow up under high concentration of ampicillin (5 mg/ml) but was recovered by the addition of 3OC6HSL (1.1 µg/ml) despite of growth retardations.

Naturally, interactions between environmental factors and ecosystem are unstable. So the structure and influent environmental factors of ecosystem fluctuated during its interplay with ecosystem. So we detected the concentration changes of ampicillin and kanamycin by LC-MS during the entire growth phase of ecosystem population (Figure S1) to better clarify biological dynamics in the ecosystem. Both antibiotics decreased with the rise up of population density due to gradual decomposition of antibiotics, but they tend to be mostly decomposed in the lag phase. The concentration of ampicillin decreased faster than kanamycin, from 4 mg/ml to 0.1 mg/ml in less than 8 hours, because the beta-lactamase coded by ampr gene can be secreted into medium to disrupt the beta-lactam ring of ampicillin. It maintained at low level(less than 0.005 mg/ml) after 12 h, but the concentration of kanamycin maintained at a higher level (0.1 mg/ml) even in the stationary phase. Kanamycin can be still detected even in stationary phase because the kanamycin was inactivated by intracellular neomycin-kanamycin phosphotransferase which phosphorylated its 3-hydroxyl group of an amino hexose instead of being degraded, and phosphotransferase cannot be secreted to medium, but the concentration of kanamycin was too low to exert influence on the ecosystem. According to our data, the dynamics of our synthetic ecosystem could be divided into several phases depending on environmental stimulus. During the lag phase, cooperation between ER and EG was much more substantial to overcome the pressure of antibiotics, thus sufficient initial cell densities were required for QS signals to initiate production of antibiotics resistance enzymes. But once the growth of ER and EG passed through the lag phase, the effects of antibiotics were weakened and competition for nutrients between two populations increased.

### Dynamics Variation depending on antibiotics concentration

Whether an ecosystem can survive typically relies on two factors: external environmental influence and inherent species composition, which were represented by antibiotics and initial cell densities respectively in our experiments. Both of them were tested by computer simulation and in vivo experiments. A mathematical model was firstly established based on ordinary differential equations (Supplementary Information S1, Table S1, Table S2) to simulate system dynamics with the addition of various levels of antibiotics.

**Figure 3:**
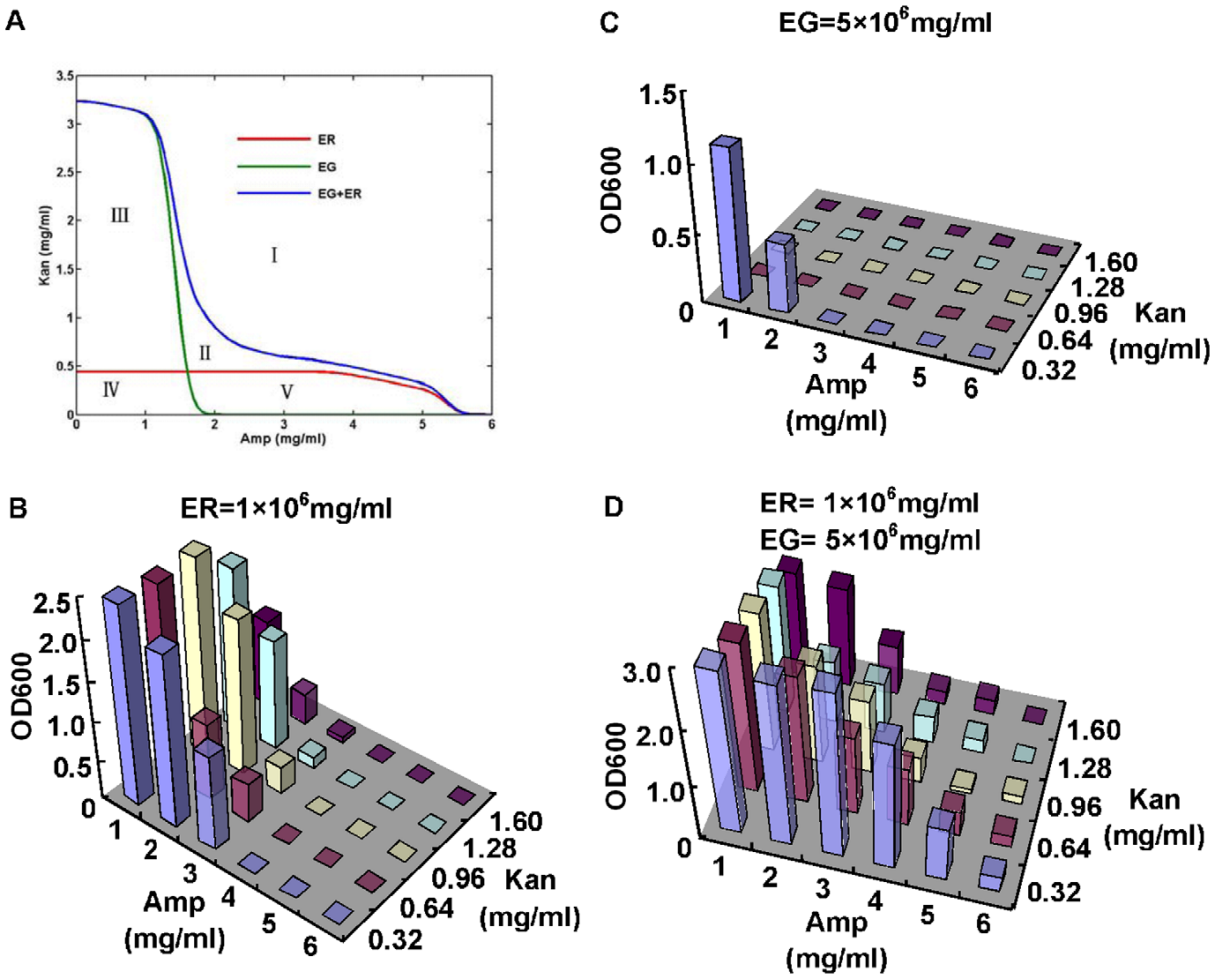
Influence of antibiotics concentration on the ecosystem population dynamics. (A) Model simulation of ecosystem status. Lines connecting bifurcation points demarcate five regions of varied biological dynamics: (I) extinction, (II) obligatory mutualism, (III) commensalism for EG, (V) facultative mutualism and (IV) commensalism for ER. (B, C, D) Population densities (OD600) of ecosystems living with different concentrations of antibiotics (growth after 24 hrs). Initial cell densities are marked on the top of each graph.

Figure 3A illustrates that with different concentrations of antibiotics, the dominating population dynamics in this ecosystem can be divided into five different types according to the changing ecological relationships between ER and EG. When antibiotic concentration reaches to a sufficiently high value, as the area I shown, ecosystem tends to extinct even with the expression of antibiotic resistance gene. Population dynamics in area II is named as obligatory mutualism area where only strictly mutualistic populations could survive. Due to the difference of ER and EG in initial antibiotic resistance, leaky expression of QS promoter and inhibition of antibiotics to cell growth, ER and EG differ in survival rates in medium with various antibiotic concentrations, which is displayed in the simulation by the area III/V where EG/ER could survive independently but ER/EG could only survive with the aid of sufficient EG/ER, a condition similar as commensalism in natural ecology. In area IV, antibiotics are not lethal for ecosystem where both ER and EG can grow independently but still benefits from each other by weakening the effects of antibiotics on cell metabolism. Additionally, competition for common nutrients is enhanced when antibiotics concentration is low. Area II in our simulation is comparatively small in our simulation, which means obligatory mutualism could only achieved in a small range of antibiotics concentration. The reason is the leaky expression of QS promoters (Supplementary Information) were big enough to enable cells to grow under low concentrations of antibiotics according to parameters provided by literatures. Whereas large amounts of antibiotics would kill too many cells to leave sufficient cells to produce QS signals and all cells would die before QS effect happens. Therefore, strict conditions are required for the existence of obligatory mutualism.

These simulation zones of the dynamic populations are further confirmed by experimental results. The sensitivity of this synthetic ecosystem to antibiotics was tested by comparing mixed culture with single strains living in mediums with various antibiotic concentration (ampicillin 1–6 mg/ml, kanamycin 0.32–1.6 mg/ml) and the same inoculation cell density (0.2×10^6^ cells/ml and 1×10^6^ cells/ml for ER and EG individually). Population densities after 24 hs' cultivation were measured and extinction was defined as the OD600 equaled to 0 and there were nearly no single colony (less than 5) appeared on LB plates spread with 100 ul fermented culture. It is shown that large amounts of antibiotics (6 mg/ml or higher ampicillin, 1.6 mg/ml or higher kanamycin) can make both population extinct no matter whether fermented separately or together. But with appropriate amounts of antibiotics, such as 4 mg/ml ampicillin, 0.64 mg/ml kanamycin, ER and EG failed to grow individually (Figure 3B, C) but can grew together (Figure 3D), which is a typical property of obligatory mutualism. Identical with our prediction, EG demonstrated less resistant to ampicillin than ER. With initial cell density 5-fold more than ER, single EG could only survive in very low antibiotics concentration compared with ER. But, one surprising phenomena is the phenotype of EG showed lower tolerance to kanamycin than ER, which may be caused by low copy number of circuit-carrying vectors in EG. Thus, the commensalism area for EG where EG could survive individually and ER could only survive with the existence of EG could not be observed. Correspondingly, commensalism for ER could be realized by keeping either of antibiotics in a suitable level (Figure 3D). When antibiotics reduced to a low level (0.16 mg/ml kanamycin, 1 mg/ml ampicillin), facultative mutualism appear to be the most important population dynamics in the ecosystem where ER and EG benefited from but not necessarily depended on each other. As Figure 3B, C and D showed, with addition of the same concentration of antibiotics, survival rates of ecosystem are obviously higher than those of individual ER and EG, especially for high antibiotics concentrations, which proved the importance of cooperation between ER and EG for their growth when exposed to environmental pressure. And one more thing worth to notice is that ER population densities largely surmount EG in all experiments, which was possibly because ER was more resistant to high antibiotic concentration than EG and it will be further investigated in the following experiments.

### Influence of initial cell densities on ecosystem status

**Figure 4:**
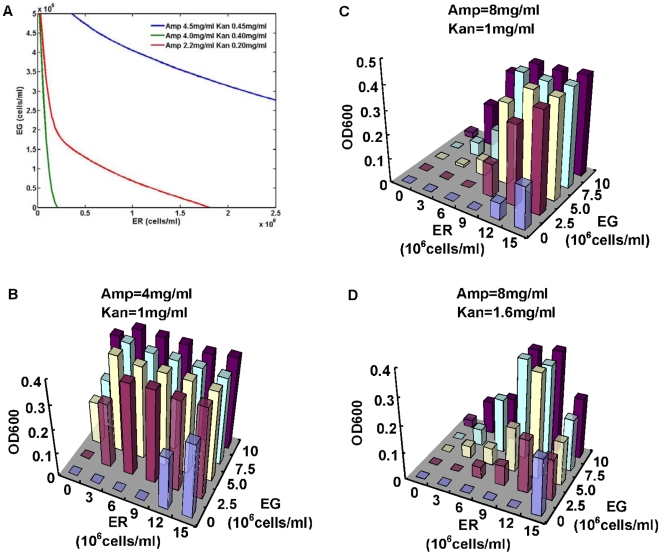
Population dynamics with various initial cell densities. (A) Model simulation of ecosystem status. The lines connect points representing minimum initial cell density required for ecosystem maintenance. (B, C, D) Population densities (OD600) after growing for 24 hrs. Initial cell densities are marked on the top of each draft.

However, the boundaries dividing ecological relations changed with initial cell densities. Generally, the higher the initial cell density is, the more possible is for cells to tolerate high level antibiotics, which is in accordance with natural ecosystems in which adequate population density is necessary for ecosystems to overcome adverse environmental influence. That is the key internal factor for our system maintenance—initial cell densities, because population dynamics in our ecosystem are based on QS promoters whose activation depends on cell densities. Simulation curves dividing the extinction area and living area (Figure 4A) show that initial cell densities have to increase with increasing antibiotics for ecosystem maintenance. For equal antibiotic concentration, EG required for ecosystem maintenance tends to be more than ER, which partially indicates EG is less tolerant to antibiotics. And our experimental results substantiate the simulation results by testing minimum initial cell densities to make ecosystems survive under different antibiotics concentration (Figure 4B, C and D). We measured the ecosystem population densities after growing cells for more than 24 hrs with different initial densities and defined the extinction as that in the last experiment. In the medium with lower antibiotic concentrations (4 mg/ml ampicillin, 1 mg/ml kanamycin), ecosystem could sustain and flourish with low initial cell density (0.5×10^6^ cells/ml ER, 0.6×10^6^ cells/ml EG) (Figure 4B). And increase of antibiotics strongly lessened stability of the ecosystem. When 8 mg/ml ampicillin and 1.6 mg/ml kanamycin were added (Figure 4D), the minimum initial cell density increased dramatically and system population density after 24 hrs' growth even decreased with higher initial cell density (as EG added from 5×10^6^ cells/ml to 7.5×10^6^ cells/ml). We also found the boundary between extinction and survival was clear, and increasing initial cell density of either ER or EG could rescue an ecosystem from extinction. However, whether an ecosystem with higher initial cell density would definitely have a higher final cell density was not confirmed, especially as Figure 4D displayed, ecosystem with 1.5×10^7^ cells/ml ER and 5×10^6^ cells/ml EG grew slower than that with 1.2×10^7^ cells/ml EG. This prove that initial cell densities only define whether ecosystem could overcome antibiotics inhibition in the beginning of growth and antibiotics were degraded too much to exert strong influence on ecosystem constitution in the later exponential and stationary phase.

### Symbiosis and competition in flasks between two populations

**Figure 5:**
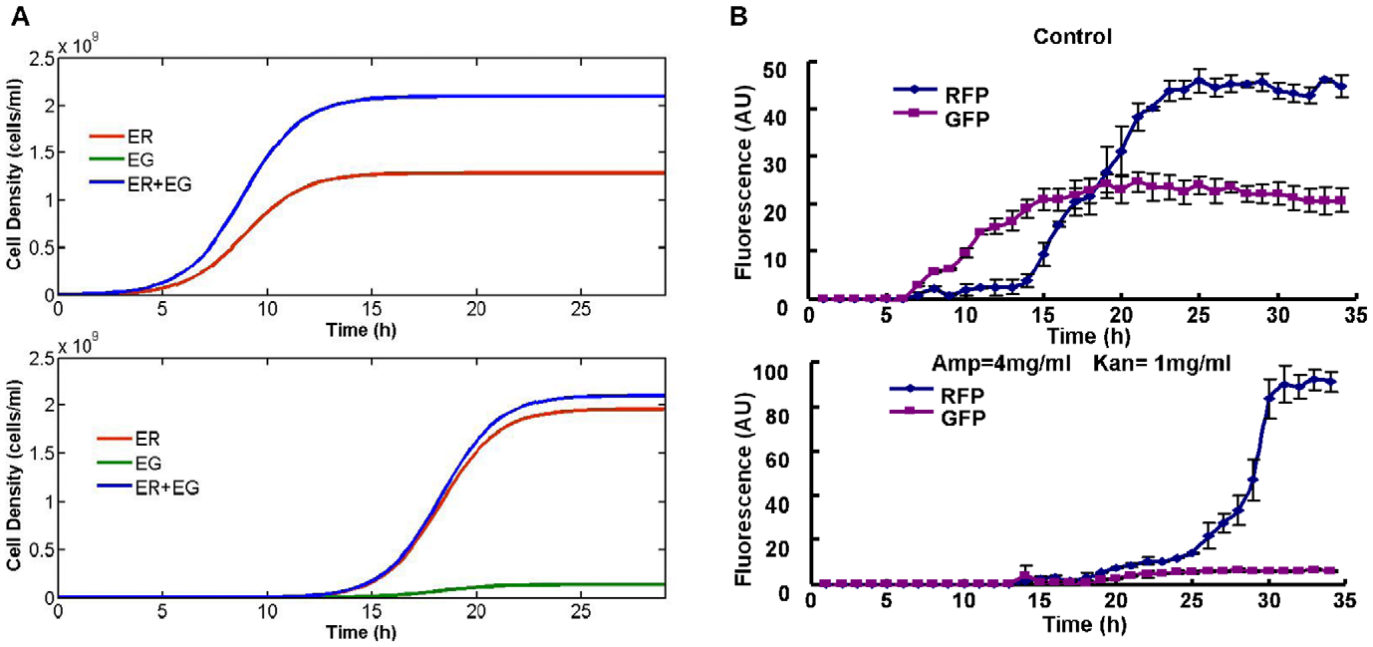
Microbial ecosystem structure difference with and without the addition of antibiotics. (A) Simulated dynamics of ER and EG cells growing with and without the pressure of antibiotics. The growth curve of ER and EG overlapped in the top graft. (B) Experimental system dynamics. The antibiotics concentration was 4 mg/ml ampicillin, 1 mg/ml kanamycin. Initial cell densities were 1×10^6^ cells/ml for ER and 1×10^7^ cells/ml for EG.

Furthermore, we simulated how co-culture competition and resistance discrimination between ER and EG affected the structure of ecosystem, assuming ER and EG shared the same capacity in nutrient assimilation, metabolism and other biological functions except for antibiotic resistance. As shown in figure 5A, the growth curves of ER and EG overlap in the medium without antibiotics, but the population equality can be broken by the addition of antibiotics which enables ER to surmount EG due to its high copy numbers of plasmids and thus more excretion of antibiotics degrading enzymes. Therefore, on one hand selective environment makes the mutualistic relationship crucial for ecosystem to prosper, so ER and EG are both necessary for the maintenance of ecosystem. On the other hand, population with higher resistance to antibiotics gets advantages in the competition for nutrients, so in the stable ecosystem, the population density of ER is nearly 7 times higher than that of EG (Figure 5A). This resistance superiority of ER to antibiotics was substantiated by growth of individual population in the medium with various high antibiotic concentrations (Figure 3B). When ampicillin and kanamycin concentrations were added with high levels (range from 1–3 mg/ml and 0.32–1.6 mg/ml respectively), ER could survive in some areas even with low initial cell density (1×10^6^ cells/ml). By contrast, even with higher initial population density (3×10^6^ cells/ml, 5×10^6^ cells/ml, 1×10^7^ cells/ml), antibiotics concentrations suitable for EG growth were limited to relatively low level (Figure 3C).

To test population ratio in co-culture, fluorescence intensity of GFP and RFP were measured by fluorescence spectrophometer. No big difference in the intensity of GFP was detected with same cell density, no matter what the antibiotics concentration was, so was RFP. Then fluorescence intensities of known population ratios were measured and the results clearly showed that the ratio of fluorescence intensity of both RFP and GFP was proportional to population ratio (Supplementary Information Figure S2). Therefore, we use fluorescence intensity as a rough representative of population ratio. In this way, different with the simulation result, ER showed greater competence than EG in the normal LB medium without antibiotics, and the population ratio of ER to EG approached 5 after 24 hr's cultivation (Figure 5B), which was possibly because genetic circuits and vectors in ER and EG have different effects on their growth. However, it was clear to find this population imbalance was exacerbated by the addition of antibiotics. Under the pressure of antibiotics, ER population overwhelmingly dominated this ecosystem and the population ratio of ER to EG was enlarged by even more than 20 times. Additionally, the initial cell density used in our experiment is 0.06×10^7^ cells/ml for ER and 0.3×10^7^ cells/ml for EG, so clearly ER became more competent in antibiotics culture.

### Conclusions

Our study verifies the influence of environmental factors on formation and diversification of a synthetic microbial ecosystem. Ecologically, environmental factors have constantly been regarded as significant elements in deciding persistence and evolution of ecosystems [36], especially for natural ecosystems which are increasingly subjected to human disruption [37], [38]. Due to the complex interactions between ecosystems and their surrounding environments, explicit empirical analyses of the evolution of interactions appear to be limited which impedes the further application and protection of microbial ecosystems. In this context, we use antibiotics as the fundamental environmental factors by changing their concentrations in a deliberately designed synthetic ecosystem. Thus, the engineered microbes have clear genetic origin and interactions for investigation and quantification.

Although sharing some similarities with previous engineering ecosystems which facilitates the elucidation of complex population dynamics and interspecies interactions of natural ecosystem, such as the predator-prey model by Arnold's group, this study is novel in considering environmental factors as key influence on ecosystem development which has been emphasized by some synthetic biologists[27]. Our research profiles the whole scenario of a synthetic ecosystem, substantiates how external factors shape population dynamics such as extinction, obligatory mutualism, facultative mutualism and commensalism, and the results suggest that transformation of population dynamics in ecosystems could be realized by altering a few major environmental factors. In addition, even though the symbiosis relationship is necessary for system maintenance, our study shows one of the two species may dominate the ecosystem, and this competence discrimination is correlated to the strength of antibiotic pressure and may make ecosystems more stable. This interesting phenomenon may help clarify how dominant species evolves in nature [39].

The ability to tolerate and recover from disturbance or stress is another important aspect of microbial ecosystem study [40], [41], and it was also investigated in our study. Here, initial population densities required for ecosystem sustenance is tightly related to antibiotic concentrations, suggesting that both environmental factors and demographic factors define survival rates and constitution of ecosystems.

Quorum sensing has exhibited sensitivity to a large range of environmental factors, which hence show great influence on microbial ecosystems utilizing QS signaling mechanism as communication methods for their components [42]. As we use the universal communication signals to build interspecies connection, our system is liable to integrate more parameters such as environmental factors or be transformed into other biological ecosystems such as competition or truly interspecies ecosystem such as yeast with bacteria [43]. If coupled with metabolic related genes, it could be possible to establish industrial ecosystem in which productivity of a valuable metabolites could be adjusted by controllable parameters.

In conclusion, the well-defined genetic background and detailed mathematical analysis of this synthetic microbial ecosystem provide us insights on how natural microbial ecosystem functions and acts as a model to illuminate complicated interactions among the environmental effects, genetic networks and system behavior.

## Materials and Methods

### Network Construction

As shown in Figure 1, two *E.coli* populations (ER and EG) are co-cultured in the medium with two antibiotics and communicate with each other through two non-interfering QS systems, LuxI/R from *Vibrio fischeri* [44] and RhlI/R from *Pseudomonas aeruginosa* [45], [46]. ER is constitutively resistant to ampicillin (resistant gene carried by vector pSB1A2), and could not express kanamycin resistant gene unless the concentration of C4HSL synthesized by RhlI in EG reaches a threshold and bind to the RhlR to activate QS promoter P_rhl_. Similarly, in EG which is constitutively resistant to kanamycin (resistant gene carried by vector pSB2K3), ampicillin resistant gene is under the control of 3OC6HSL produced by ER. Therefore, when cultured in the medium containing ampicillin and kanamycin, ERs and EGs cells require mutualistic interactions between them to grow. However, low levels of antibiotics make mutualism dispensable for system maintenance and mutualism is unable to rescue populations growing with extremely high levels of antibiotics, so the development of this ecosystem depends on antibiotics concentrations, which are the key environmental factors in our study. All biobricks used in our experiments are listed in Table S3.

Several other factors also play roles in shaping system dynamics. First, luxPR and P_rhl_ promoters employed in our experiment had leaky expressions without combination of signal-transcriptional regulator, a feature of natural QS system [47]. Thus, ER and EG could tolerate low concentrations of kanamycin and ampicillin respectively, and the system turned to be facultative mutualism in which both species did not strictly need the existence of the other to survive. Second, ER and EG showed divergence in their resistance to high concentrations of antibiotics, which could be contributed to their different resistance gene expressions. Plasmids used in experiments include pSB1A2 (pMB1 origin of replication with a copy number of 100–300 per cell) for ER and pSB2k3 (F' origin of replication with a copy number of less than 10 per cell) for EG. Thus, with sufficient molecule signals and identical antibiotic concentration, the antibiotic resistance of ER is far more than that of EG, thus created population imbalance. Besides, there exists co-culture competition between ER and EG for common nutrients such as carbon sources. Although in computer simulation we assumed their competence for nutrients was the same due to their origin of the same strain, diverse properties of colonies could lead to different experimental results. In addition, to distinct ER and EG for experimental detection, they were labeled with red-fluorescence protein (RFP) and green-fluorescence protein (GFP) separately.

### Cell Density Measurement

Total population densities of ecosystem was measured by spectrophotometer (722, shanghai). ER and EG were inoculated overnight in LB medium to stationary phase when fluorescence intensity (a.u.) of RFP and GFP kept stable, then the culture was diluted five times to make sure OD600 is between 0.0–0.7. Then values of OD600 were used to calculate total population densities (1 OD equals to around 10^9^ cells/ml).

Individual cell densities of ER and EG were measured by fluorescence spectrophotometer. The mixed cells of ER and EG were cultured to stationary phase (Cell density approximate to 2×10^9^ cells/ml) in the LB medium with antibiotics. Then fermented culture was diluted five times with fresh LB medium and fluorescence of GFP and RFP were measured by fluorescence spectrophotometer (Varian, Cary Eclipse) with exciting wavelength of 501 nm, emitting wavelength of 511 nm for GFP, and exciting wavelength of 584 nm, emitting wavelength of 607 nm for RFP. The fluorescence intensity of fresh LB medium was defined as blank control. All-wavelength scanning showed the absorbance peak of GFP had very little overlap with that of RFP and so the intensity of GFP fluorescence approached zero under the condition that exciting wavelength equaled to 584 nm, emitting wavelength equaled to 607 nm. The fluorescence intensity of cells without expression of GFP and RFP approached zero during the whole growing process (data not shown), and the fluorescence intensities of GFP and RFP in different culture tended to fluctuate in a small scale. Thus fluorescence intensities of GFP and RFP could be used to calculate cell density of EG and ER respectively. Through mixing different concentrations of EG and ER together, a linear relationship between fluorescence intensity and cell density was established by which cell densities of ER and EG living with various concentrations of antibiotics could be calculated by fluorescence intensities of GFP and RFP (Figure S2).

### Strains, Growth Conditions

DH5α *E.coli* cell strains (Progen, China) were used as fundamental engineering chassis. For single cell function test, we used 3OC6HSL (Sigma Aldrich) and C4HSL (Sigma Aldrich), which were prepared and stored as provided instructions. LB medium were used for liquid culture. Antibiotics (ampicillin and kanamycin) were dissolved with distillation water and stored as 100 mg/ml and 50 mg/ml respectively, whose concentrations in the medium depended on experiments. The seed cultures of ER and EG were inoculated from glycerol stock solution and grown separately overnight, and then diluted to about 1000 times into 5 ml of fresh medium, which were cultured at 37°C with a shaking rate of 220 r.p.m.

### Microscopic Cell Imaging

Overnight-cultured ER and EG medium were collected and diluted 1000 times before microscopic observation. Fluorescence intensities of GFP and RFP were detected by fluorescence microscope (Nikon) with the exciting wavelength from 450 to 490 nm and 510–560 nm respectively.

### LC-MS Detection of Antibiotics

The experiment was performed on a Finnigan LC–MS/MS system (Thermofisher Scientific, San Jose, CA, USA) consisting of Surveyor HPLC with quaternary gradient pumps and an autosampler coupled with LCQ Advantage Max ion-trap mass spectrometer.

Kanamycin and ampicillin were separated on a Symmetry C18 column (150 mm×2.1 mm, 5 µm, Waters). Mobile phase A was water (0.1% formic acid, v/v) and mobile phase B was acetonitrile (0.1% formic acid, v/v). The antibiotics were eluted isocratically with 20% B for 15 min at the flow rate of 200 µl·min^−1^. The injection volume was 5 ul.

LC–MS/MS conditions were: ESI spray voltage, 5 kV; Nitrogen was used as sheath and auxiliary gases with flow rates of 35 and 5 units, respectively; capillary temperature, 300°C and tube lens, 30 V. The SRM scan in positive ionization mode was used for the determination of two compounds. Kanamycin and Ampicillin were monitored at *m*/*z* transitions of 485.1→324.0, 350.0→160.0 with the normalized collision energies for 25% and 30% respectively.

## Supporting Information

Figure S1 Degradation of antibiotics within symbiosis system cultured for 24 hours. With the increase of population density, ampicillin level falls down from 4 mg/ml to 0.1 mg/ml within 8 hs and maintains low level(less than 0.005 mg/ml) after 12 h. Knamycin level falls from 1 mg/ml to 0.1 mg/ml within 14 hs and maintains 0.1 mg/ml after 14 h.
(0.10 MB TIF)

Figure S2 Levels of fluorescence intensity of various population ratios. EG and ER are mixed in according to definite ratio, with the total density is 109 cells/ml. Their fluorescence intensity is measured by fluorescence spectrophotometer and are also converted into fraction of EG's. The curve is almost linear and shows the fluorescence intensity of both RFP and GFP is proportional to population ratio.
(0.09 MB TIF)

Table S1 State variables and parameters of the model.
(0.05 MB DOC)

Table S2 Parameter values.
(0.04 MB DOC)

Table S3 Biobricks used in the experiments.
(0.03 MB DOC)

Supplementary Information S1 (0.08 MB DOC)
