# Supplementary material for: An Environment-Sensitive Synthetic Microbial Ecosystem
Source: PLoS One. 2010 May 12;5(5):e10619. doi: 10.1371/journal.pone.0010619 (PMC2868903; doi:10.1371/journal.pone.0010619)
Supplement: Table S1 — State variables and parameters of the model. (0.05 MB DOC) [file pone.0010619.s003.doc]

**Table S1.** State variables and parameters of the model

| **Variable** | **Description** | **Unit** |
| --- | --- | --- |
| N_ER_ | Cell Density of ER | OD 600 |
| N_EG_ | Cell Density of EG | OD 600 |
| C_amp_ | Ampicillin Concentration | g/L |
| C_kan_ | Kanamycin Concentration | g/L |
| 3OC_6_HSL | Concentration of 3OC_6_HSL Signal | nM |
| C_4_HSL | Concentration of C_4_HSL Signal | nM |
| G_ER_ | Average concentration of resistance enzyme in per ER cell | μM |
| G_EG_ | Average concentration of resistance enzyme in per EG cell | μM |
| **Parameters** | **Description** | **Unit** |
| N_m_ | Maximum cell density | OD 600 |
| γ_amp_ | Antibiotic inhibition constant for ampicillin | L/(g. h) |
| γ_kan_ | Antibiotic inhibition constant for kanamycin | L/(g. h) |
| resistR | Native ampicillin resistance enzymes concentration in ER | μM |
| resistG | Native kanamycin resistance enzymes concentration in EG | μM |
| μ_ER_ | Cell growth rate constant for ER | 1/h |
| μ_EG_ | Cell growth rate constant for EG | 1/h |
| η_amp_ | Effect constant of ampicillin resistance gene to ampicillin | g/(L^.^μM) |
| η_kan_ | Effect constant of kanamycin resistance gene to kanamycin | g/(L^.^μM) |
| d_anti_ | Degradation rate of antibiotic | 1/(h^.^OD) |
| k_3OC6HSL_ | Production rate of 3OC6HSL molecular | nM/(h^.^OD) |
| k_C4HSL_ | Production rate of C4SHL molecular | nM/(h^.^OD) |
| d_3OC6HSL_ | Degradation rate of 3OC6HSL molecular | 1/h |
| d_C4HSL_ | Degradation rate of C4SHL molecular | 1/h |
| α_ER_ | Max production rate of kanamycin resistance enzyme in ER | μM/h |
| α_EG_ | Max production rate of ampicillin resistance enzymes in EG | μM/h |
| β | Hill coefficient for kanamycin resistance gene in ER |  |
| γ | Hill coefficient for ampicillin resistance gene in EG |  |
| m_ahl_ | Hill parameter for kanamycin resistance gene in ER | nM |
| m_bhl_ | Hill parameter for ampicillin resistance gene in EG | nM |
| leakyR | Leaky expression rate of kanamycin resistance gene in ER | μM/h |
| leakyG | Leaky expression rate of ampicillin resistance gene in EG | μM/h |
| d_ER_ | Degradation rate of kanamycin resistance gene in ER | 1/h |
| d_EG_ | Degradation rate of ampicillin resistance gene in EG | 1/h |
