# Supplementary material for: An Environment-Sensitive Synthetic Microbial Ecosystem
Source: PLoS One. 2010 May 12;5(5):e10619. doi: 10.1371/journal.pone.0010619 (PMC2868903; doi:10.1371/journal.pone.0010619)
Supplement: Table S2 — Parameter values. (0.04 MB DOC) [file pone.0010619.s004.doc]

**Table S2.** Parameter values

| **Parameter** | **Value** | **Method** | **Parameter** | **Value** | **Method** |
| --- | --- | --- | --- | --- | --- |
| N_m_ | 2.8 OD 600 | a | d_3OC6HSL_ | 0.6 /h | c |
| γ_amp_ | 0.19 L/(g. h) | d e g | d_C4HSL_ | 0.6 /h | c |
| γ_kan_ | 1.9 L/(g. h) | de g | α_ER_ | 80 μM/h | c f g |
| resistR | 20 μM | d f g | α_EG_ | 40 μM/h | c f g |
| resistG | 10 μM | d f g | β | 1.6 | c |
| μ_ER_ | 0.082 /h | a | γ | 1.6 | c |
| μ_EG_ | 0.082 /h | a | m_ahl_ | 1.5 nM | c |
| η_amp_ | 0.5 g/(L^.^μM) | d e g | m_bhl_ | 1.5 nM | c |
| η_kan_ | 0.05 g/(L^.^μM) | d e g | leakyR | 5 μM/h | d f g |
| d_anti_ | 20 /(h^.^OD) | b | leakyG | 2.5 μM/h | d f g |
| k_3OC6HSL_ | 20 nM/(h^.^OD) | c f g | d_ER_ | 4.0 /h | c |
| k_C4HSL_ | 10 nM/(h^.^OD) | c f g | d_EG_ | 4.0 /h | c |
