# Supplementary material for: An Environment-Sensitive Synthetic Microbial Ecosystem
Source: PLoS One. 2010 May 12;5(5):e10619. doi: 10.1371/journal.pone.0010619 (PMC2868903; doi:10.1371/journal.pone.0010619)
Supplement: Table S3 — Biobricks used in the experiments. (0.03 MB DOC) [file pone.0010619.s005.doc]

**Table S3.** Biobricks used in the experiments

| **Names** | **Code** | **Length** | **URL** |
| --- | --- | --- | --- |
| *RFP* | I13521 | 923bp | http://partsregistry.org/Part:BBa_I13521 |
| *RhlR* | I1466 | 884bp | http://partsregistry.org/Part:BBa_I1466 |
| *RhlI* | I0405 | 1633bp | http://partsregistry.org/Part:BBa_I0405 |
| *3OC6HSL receive (LuxR)* | F2620 | 1061bp | http://partsregistry.org/Part:BBa_F2620 |
| *Rhl Promoter* | R0071 | 53bp | http://partsregistry.org/Part:BBa_R0071 |
| *Ptet* | R0040 | 54bp | http://partsregistry.org/Part:BBa_R0040 |
| *LuxI* | I13202 | 803bp | http://partsregistry.org/Part:BBa_I13202 |
| *GFP* | I13522 | 937bp | http://partsregistry.org/Part:BBa_I13522 |
| *RBS* | B0034 | 12bp | http://partsregistry.org/Part:BBa_B0034 |
| *Terminator* | B0015 | 129bp | http://partsregistry.org/Part:BBa_B0015 |
