# Supplementary material for: An Environment-Sensitive Synthetic Microbial Ecosystem
Source: PLoS One. 2010 May 12;5(5):e10619. doi: 10.1371/journal.pone.0010619 (PMC2868903; doi:10.1371/journal.pone.0010619)
Supplement: Supplementary Information S1 — (0.08 MB DOC) [file pone.0010619.s006.doc]

**An Environment Sensitive Synthetic Microbial Ecosystem**

**SUPPLEMENTARY INFORMATION**

## Model Development

In order to understand the behavior of our synthetic microbial ecosystem, a mathematical model with ODEs (equation S1-S6) is used to simulate the system under different conditions. The state variables and parameters are described in detail in Table S1.

(S3)

(S4)

(S5)

(S6)

(S7)

(S8)

(S1)

(S2)

The major kinetic events in the ecosystem that determine the behavior are: population growth (S1, S2); antibiotic degradation by resistance enzymes (S3, S4); QS signal molecular synthesis and degradation (S5, S6) and resistance gene expression under the control of QS promoters (S7, S8). All together, the interaction of 8 key variables (the cell number of EG and ER, the concentrations of two antibiotics, two QS signal molecular and two controlled resistance enzymes) decide the performance of the ecosystem. Following words describe how dynamic equations are developed.

Free growth of two cells

The growth state of individual cell without effect of antibiotics can be described by logistic growth law (S9) [1] , in which, N represents the cell number in system and Nm represent the maximum allowed number of cells due to nutrient limitation, k is a constant coefficient.

$dN/dt=k\cdot N\cdot(1-N/N_{m})$ (S9)

The growth rate is proportional to cell number (k·N) and inhibited by themselves due to the limitation of nutrients (1-N/Nm). The growth of mixed two populations (N1, N2) with similar properties, like ER and EG (both E.coli DH5α strains with similar genetic manipulation), can be described as S10 and S11. The growth rate of any kind of the cells is mixed to its own population (k1·N1 or k2·N2) but inhibited by total population of cells (1-(N1+N2)/Nm). It’s easy to prove the equation by adding S10 and S11 to form S12 which is the same as S9 (N=N1+N2).

$dN_{1}/dt=k_{1}\cdot N_{1}\cdot(1-(N_{1}+N_{2})/N_{m})$ (S10)

$dN_{2}/dt=k_{2}\cdot N_{2}\cdot(1-(N_{1}+N_{2})/N_{m})$ (S11)

$d(N_{1}+N_{2})/dt=k\cdot(N_{1}+N_{2})\cdot(1-(N_{1}+N_{2})/N_{m})$ (S12)

The inhibition of antibiotics to E.coli

The inhibition of antibiotics is represented simply by equation S13, where C is the antibiotic concentration and γ is the rate constant. Both the antibiotic concentration in the medium and the number of cells that exposed to the antibiotic contribute to the decreasing rate.

$dN/dt=-\gamma\cdot N\cdot C$ (S13)

The expression of antibiotic resistance gene could release the antibiotic inhibition to some extent according to the synthesized enzyme concentration. So S14, in which resist is the expression level of antibiotics gene and η is constant for specific gene, can replace S13. If (C-η·resist) is negative which means that the resistance enzyme is more than enough, this term is set to zero while simulation since the maximum effect of the resistance enzyme is to eliminate all the effect of antibiotic.

$dN/dt=-\gamma\cdot N\cdot(C-\eta\cdot resist)$ (S14)

Therefore, the dynamic equations of the two cells’ growth are listed as S1 and S2. The cell number can be replaced by cell density (OD 600) since the total volume of the system is fixed in all experiments.

Antibiotic degradation by resistance enzymes

Antibiotics are the key environmental factors that largely define system behavior and population dynamics. In our design, antibiotics were added at the beginning of fermentation and gradually degraded due to the synthesis of resistance enzymes. The natural degradation can be neglected since enzymes are much faster. The degradation process can be simplified to S15, where d is the degradation rate and Ni·resisti·η represents the contributions from different kind of cells. As described before, η is the parameter connecting the level of degrading enzyme and antibiotics concentration, N is total cell density and resist is the resistance enzyme concentration.

$dC/dt=-d\cdot\sum(N_{i}\cdot resist_{i}\cdot\eta)$ (S15)

So the variation of antibiotics concentration is displayed by equation S3 and S4. resistR and resistG are the concentration of the resistance enzymes that natively expressed in the cells and we assume that they are invariable during the process. Making the assumption that the better the resistance gene can rescue the more the antibiotic is degraded, the degrading of different antibiotics are related to the rescue parameter η and the degrading rate constants (danti) are the same.

QS signal molecular synthesis and degradation

The QS-signal molecules constitutively expressed under the control of Ptet promoter and would be gradually degraded in culture medium. So the synthesis rate of signal is proportional to cell density and the degradation rate is proportional to molecule concentration. S5, S6 are generated based on this assumption.

Resistance gene expression under the control of QS promoters

The level of resistance enzyme is control by a complex process which includes the combination of signals to related inducer, initialization of transcription and translation, and the degradation of protein. As constitutively expressed proteins, the level of LuxR and RhlR is supposed to be fixed in single cell. So the expression of antibiotic genes could be generated by Hill function (S16) according to the concentration of QS-signal molecular. Hill function [2], in which L means the ligand (QS-signal molecules in this model), α is the max expression speed of the promoter, β is Hill coefficient and m represent the ligand concentration producing half occupation, is widely used in synthetic biology to simulate genetic circuit performance. Together with leakyR and leakyG that represent the leaky expression of the resistance genes which is observed in the experiment and the degradation of the enzymes in the cell, S7 and S8 are used to simulate the expression of resistance genes per cell.

$dG/dt=\alpha\cdot L^{\beta}/(m^{\beta}+L^{\beta})$ (S16)

## Parameters

The parameters are listed in the Table S1 and Table S2. It takes several steps to determine parameter values (the method letters indicate how the parameters are obtained):

1. Some values are calculated from experiment: the cell growth curve (a) and LC-MS detection of antibiotics (b).

2. The parameters for QS-signal molecule production and controlling of QS-promoters are borrowed from literatures [3-5] with the similar simulation (c).

3. The parameters regarding to the inhibition of growth by the antibiotics and succor by the resistance enzymes are estimated based on the extreme condition of dynamic relationship(Figure 3, Figure 4) (d), such as with only one kind of antibiotic or cell.

4. Based on the experiment, the ratio of some parameters are fixed. The potency of kanamycin to the ampicillin is 10:1 (e) and the ratio of plasmid copy number in ER to that in EG is 2:1 (f).

5. Finally, some of the parameters are further optimized to fit the result of growth curve of mixed ecosystem (Figure 5) (g).

## References
